# Wheat germ-based protein libraries for the functional characterisation of the Arabidopsis E2 ubiquitin conjugating enzymes and the RING-type E3 ubiquitin ligase enzymes

**DOI:** 10.1186/s12870-015-0660-9

**Published:** 2015-11-10

**Authors:** Abdelaziz Ramadan, Keiichirou Nemoto, Motoaki Seki, Kazuo Shinozaki, Hiroyuki Takeda, Hirotaka Takahashi, Tatsuya Sawasaki

**Affiliations:** Proteo-Science Center, Ehime University, Matsuyama, 790-8577, Japan; Plant Genomic Network Research Team, RIKEN Center for Sustainable Resource Science, 1-7-22 Suehiro-cho, Tsurumi-ku, Yokohama, Kanagawa 230-0045, Japan; Gene Discovery Research Group, RIKEN Center for Sustainable Resource Science, 1-7-22 Suehiro-cho, Tsurumi-ku, Yokohama, Kanagawa 230-0045, Japan; Botany Department, Faculty of Science, Ain Shams University, Cairo, 11566, Egypt; CREST, Japan Science and Technology Agency (JST), 4-1-8 Honcho, Kawaguchi, Saitama 332-0012, Japan

**Keywords:** *Arabidopsis thaliana*, Ubiquitination, Ubiquitin-conjugating enzymes, RING-type Ubiquitin ligase enzymes, Wheat germ-based protein libraries, E2/E3 screening

## Abstract

**Background:**

Protein ubiquitination is a ubiquitous mechanism in eukaryotes. In Arabidopsis, ubiquitin modification is mainly mediated by two ubiquitin activating enzymes (E1s), 37 ubiquitin conjugating enzymes (E2s), and more than 1300 predicted ubiquitin ligase enzymes (E3s), of which ~470 are RING-type E3s. A large proportion of the RING E3’s gene products have yet to be characterised *in vitro*, likely because of the laborious work involved in large-scale cDNA cloning and protein expression, purification, and characterisation. In addition, several E2s, which might be necessary for the activity of certain E3 ligases, connot be expressed by *Escherichia coli* or cultured insect cells and, therefore, remain uncharacterised.

**Results:**

Using the RIKEN Arabidopsis full-length cDNA library (RAFL) with the ‘split-primer’ PCR method and a wheat germ cell-free system, we established protein libraries of Arabidopsis E2 and RING E3 enzymes. We expressed 35 Arabidopsis E2s including six enzymes that have not been previously expressed, and 204 RING proteins, most of which had not been functionally characterised. Thioester assays using dithiothreitol (DTT) showed DTT-sensitive ubiquitin thioester formation for all E2s expressed. In expression assays of RING proteins, 31 proteins showed high molecular smears, which are probably the result of their functional activity. The activities of another 27 RING proteins were evaluated with AtUBC10 and/or a group of different E2s. All the 27 RING E3s tested showed ubiquitin ligase activity, including 17 RING E3s. Their activities are reported for the first time.

**Conclusion:**

The wheat germ cell-free system used in our study, which is a eukaryotic expression system and more closely resembles the endogenous expression of plant proteins, is very suitable for expressing Arabidopsis E2s and RING E3s in their functional form. In addition, the protein libraries described here can be used for further understanding E2-E3 specificities and as platforms for protein-protein interaction screening.

**Electronic supplementary material:**

The online version of this article (doi:10.1186/s12870-015-0660-9) contains supplementary material, which is available to authorized users.

## Background

Protein ubiquitination is a posttranslational modification involving a highly conserved 76-amino acid protein called ubiquitin (Ub), which regulates a multitude of targets in eukaryotes [1–3]. In plants, ubiquitination is involved in the regulation of various biological processes including growth and development, response to biotic and abiotic stress signalling, and regulation of chromatin structure [4–7]. The covalent attachment of Ub to a diverse array of target proteins requires a cascade of reactions catalysed by three kinds of enzymes: ubiquitin-activating enzyme (E1), ubiquitin-conjugating enzyme (E2), and ubiquitin ligase enzyme (E3). E3s are the most diverse enzymes in the ubiquitination cascade and are probably the main determinant of substrate specificity [2]. E3 proteins are classified into three classes according to the presence of one of the following domains: homology of the E6-AP C-terminus (HECT), U-box, or really interesting new gene (RING). These domains act mainly as E2 docking sites.

Ubiquitination is initiated by E1-dependent activation of Ub in an ATP-dependent reaction, ultimately forming a thioester linkage between an E1 catalytic Cys residue and the carboxyl-terminal Gly of Ub. This activated Ub is then transferred via thioester linkage to a catalytic Cys residue within the UBC domain of E2. Finally, E3 proteins identify the target protein and mediate formation of an isopeptide bond between the C-terminal Gly carboxyl group of Ub and a target Lys ε-amino group. Depending on the type of E3, Ub transfer to the target protein in the final step occurs directly from the E2 (RING- and U-box-type E3s) or after thioester formation of Ub with the E3 (HECT-type E3s) [6]. The outcomes of the E1-E2-E3 enzymatic reactions vary greatly since they may add one or more Ub(s) to the target protein (monoubiquitination or polyubiquitination, respectively) in different configurations [8]. Consequently, ubiquitination can act as a signal for protein activation, degradation by the 26S proteasome, intracellular localization, vesicular trafficking, or histone modification and transcription regulation [9, 10].

In Arabidopsis, the genes encoding the enzymes that mediate Ub modification represent a significant fraction of the genome [2]. Two related genes encode E1 in the Arabidopsis genome, UBIQUITIN ACTIVATING 1 (AtUBA1) and UBIQUITIN ACTIVATING 2 (AtUBA2) [11]. These proteins share about 80 % amino acid identity with each other, as well as conserved amino acid sequences with mammalian and yeast enzymes [8]. In the case of E2s, the Arabidopsis genome encodes 48 proteins that contain a conserved region of approximately 140–200 amino acids, called the UBC domain [12, 13]. Thirty-seven of 48 UBC domain-containing proteins are thought to conjugate to Ub (E2s). Another eight lack the catalytic Cys called ubiquitin enzyme variants (UEVs), and the remaining three catalyse the conjugation of ubiquitin-like proteins (UBLs). The 48 UBCs have been classified into 16 subgroups according to their identity with each other [13]. For E3s, more than 1300 genes are predicted to encode E3 ligase components in the Arabidopsis genome [2]. The HECT and U-box domain-containing proteins are encoded by seven and 64 genes, respectively [6], while more than 470 genes encode the RING domain-containing proteins [14]. The E3s that utilize the RING domain for E2 binding can be subdivided into simple and complex E3s. In many cases, the simple RING E3s contain both the E2 binding domain (RING) and the substrate binding domain within a single protein, whereas in other cases, they may act as homo or heterodimers of two different RING proteins [14]. On the other hand, the complex RING E3s contain multiple different proteins. Best characterized are the cullin-RING ligase (CRL) E3s, consisting, in Arabidopsis, of CULLIN1, CUL3a/b or CUL4, which serve as a platform linking one of two closely related RING-type proteins (RBX1a/b) to one of over 800 substrate-recognition subunits [15]. For ease of *in vitro* characterisation, in our study, we focused on the simple Arabidopsis RING E3s.

The RING-type E3 ligases share a Cys-rich RING domain that contains eight conserved Cys and/or His residues and binds two Zinc (Zn) ions [14, 16]. Some other domains, such as the Zn finger, LIM, and PHD, also showed similar patterns of Cys and His residues as found in the RING domain, although they differ in their folding and function [17, 18]. The eight Zn-coordinating residues in the RING domain form a cross-brace structure with Zn ions, which acts as a platform for E2 interaction [16]. The Arabidopsis RING proteins were classified into three RING types (RING-H2, RING-HCa, and RING-HCb) and five modified RING types (RING-v, RING-C2, RING-D, RING-S/T, and RING-G) based on the type of Zn-coordinating residues and the number of amino acids between them [14]. Mutations in one or more of these Zn-coordinating residues may disrupt the RING domain to mediate protein ubiquitination.‬‬‬‬‬‬‬‬‬‬‬‬‬‬‬‬‬‬‬

As the requirements of RING E3s activity *in vitro* are believed to be identical to those *in vivo*, even in the absence of their physiological substrates [13], functional characterization of gene products of RING E3s is possible. To our knowledge, the largest scale analysis performed previously utilised proteins expressed in *E. coli* cells. Ubiquitination activity of ~64 RING E3 ligases was first tested *in vitro* with AtUBC8 [14], then with representative members of different UBC subfamilies [13]. Whereas the majority of RING E3s tested showed activity, 19 RING E3s showed no activity with all E2s tested [13, 14]. Seven E2s were insoluble after expression using *E. coli* and/or cultured insect cells [13], preventing their utilization in ubiquitination assays. It is possible that one of these E2s is required for the activity of these apparently inactive E3s, the E3s were expressed with improper folding or additional proteins are required. Therefore, in our study we used a eukaryotic cell-free system to express and analyse the activity of Arabidopsis E2s and RING E3s. Biochemical characterisation of gene products using cell-free protein synthesis systems is very convenient because cellular toxicity is not a concern [19]. In particular, the wheat germ cell-free system, which is a eukaryotic expression system and more closely resembles endogenous expression of plant proteins showed successful expression of several multi-domain eukaryotic proteins in functional form [20]. For large-scale analysis of Arabidopsis E2s and RING E3s, we used the RIKEN Arabidopsis full-length (RAFL) cDNA library as the main source of E2s and RING E3s cDNAs. Using the ‘split-primer’ PCR method for the high-throughput preparation of transcription templates and the wheat germ cell-free system, we constructed protein libraries including 35 E2s and 204 RING E3s. Finally, we demonstrated biochemical activity for all E2s expressed and for representative RING E3s using wheat germ crude extracts.

## Results

### The wheat germ cell-free system expressed 35 of the 37 Arabidopsis E2s

**Fig. 1:**
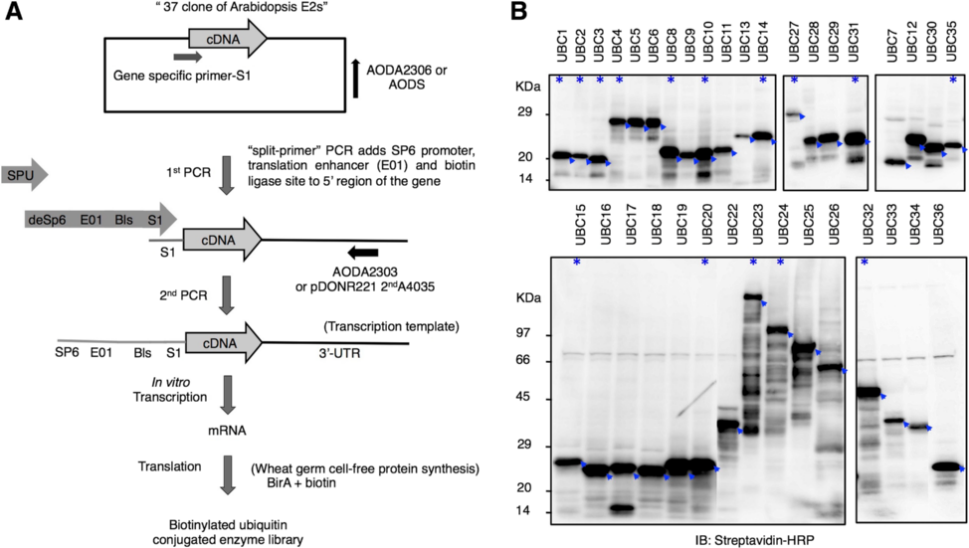
Construction of an Arabidopsis E2 protein library with an N-terminus biotin tag using a wheat germ cell-free system. **a** Flow chart of the wheat germ-based procedure for the high-throughput production of an Arabidopsis E2 library with an N-terminus biotin tag. The first step involves the high-throughput preparation of DNA templates for transcription using 2-step “split-primer” PCR, followed by *in vitro* transcription using phage-coded SP6 RNA polymerase, and finally translation using the wheat germ cell-free system. All the steps were carried out in 96-well microtiter plates. **b** Immunoblot analysis of N-bio-E2s expressed by the wheat germ cell-free system. For analysis, 2–6 μL crude recombinant E2 proteins with N-terminus biotin tag were loaded onto SDS-PAGE and detected by streptavidin-HRP antibody. A total of 35 out of 37 predicted Arabidopsis E2s were detected. Arrows on the figure show the expected signal for each E2 and asterisks refer to the E2s used later *in vitro* ubiquitination analysis (Fig. 4, Fig. 5, Fig. 6)

We aimed to collect as many cDNA clones as possible for E2s that are currently annotated in Arabidopsis in order to express them using wheat germ cell-free system and to test their functional activity. The Arabidopsis genome is predicted to encode 37 genes thought to function as E2s [13]. We collected the cDNA clones for these 37 genes either from RAFL cDNA library [21] or from other resources outlined in Table 1. Using the ‘split-primer’ PCR and the 37 cDNA clones as templates, we prepared the transcription templates by adding the sequences of the SP6 promoter, E01 enhancer region, and Biotin ligase site (Bls) to the 5’-end (Fig. 1a). This method is suitable for high-throughput preparation of transcription templates [22]. *In vitro* transcription followed by translation by the bilayer mode of wheat germ cell-free system surprisingly yielded the expression of 35 N-terminal biotinylated (N-bio-) E2s from the 37 genes (Fig. 1b). This expression analysis represents the largest collection of translated Arabidopsis E2s compared with previous studies. A group of E2s including UBC12, UBC23, UBC24, UBC25, UBC31, and UBC33, which had not previously been expressed *in vitro* [8, 13, 23], was successfully expressed using the wheat germ cell-free system. Only the UBC21 and UBC37 proteins were not expressed using our expression system. Their mRNA level was comparable to others, but we could not detect the corresponding protein by immunoblotting analysis. UBC37 was reported to undergo extensive proteolysis when expressed in bacteria [13], whereas UBC21 was not expressed when either *E. coli* or cultured insect cells were used [13, 23].

**Table 1:** Summary of Arabidopsis E2s used in this study, their protein expression in a wheat germ cell-free system and activity as E2 enzymes

Gene name	AGI loci	Other names	Subfamily	M. wt. (Da)	Protein expression	Thioester formation
UBC1	At1g14400^(a)^		III	17,280	Yes	Yes
UBC2	At2g02760^(a)^		III	17,270	Yes	Yes
UBC3	At5g62540^(a)^		III	17,130	Yes	Yes
UBC4	At5g41340^(a)^		IV	21,300	Yes	Yes
UBC5	At1g63800^(a)^		IV	19,400	Yes	Yes
UBC6	At2g46030^(a)^		IV	20,890	Yes	Yes
UBC7	At5g59300^(b)^		V	22,900	Yes	Yes
UBC13	At3g46460^(a)^		V	18,820	Yes	Yes
UBC14	At3g55380^(a)^		V	18,720	Yes	Yes
UBC8	At5g41700^(a)^		VI	16,530	Yes	Yes
UBC9	At4g27960^(a)^		VI	16,550	Yes	Yes
UBC10	At5g53300^(a)^		VI	16,530	Yes	Yes
UBC11	At3g08690^(a)^		VI	16,550	Yes	Yes
UBC12	At3g08700^(c)^		VI	16,710	Yes	Yes
UBC28	At1g64230^(a)^		VI	19,270	Yes	Yes
UBC29	At2g16740^(a)^		VI	16,760	Yes	Yes
UBC30	At5g56150^(a)^		VI	16,480	Yes	Yes
UBC15	At1g45050^(a)^	ATUBC2-1	VII	18,260	Yes	Yes
UBC16	At1g75440^(a)^		VII	18,490	Yes	Yes
UBC17	At4g36410^(b)^		VII	18,670	Yes	Yes
UBC18	At5g42990^(a)^		VII	18,370	Yes	Yes
UBC19	At3g20060^(a)^		VIII	20,000	Yes	Yes
UBC20	At1g50490^(a)^		VIII	21,460	Yes	Yes
UBC21	At5g25760^(b)^	PEX4	IX	17,710	No	nd
UBC22	At5g05080^(a)^		X	27,400	Yes	Yes
UBC23	At2g16920^(b)^	PFU2	XI	122,190	Yes	Yes
UBC24	At2g33770^(a)^	PHO2	XI	100,490	Yes	Yes
UBC25	At3g15355^(a)^	PFU1	XI	67,780	Yes	Yes
UBC26	At1g53025^(a)^	PFU3	XI	60,554	Yes	Yes
UBC27	At5g50870^(a)^		XII	21,250	Yes	Yes
UBC31	At1g36340^(b)^		XIII	17,830	Yes	Yes
UBC32	At3g17000^(a)^		XIV	34,320	Yes	Yes
UBC33	At5g50430^(a)^		XIV	27,360	Yes	Yes
UBC34	At1g17280^(a)^		XIV	26,610	Yes	Yes
UBC35	At1g78870^(b)^	UBC13A	XV	17,190	Yes	Yes
UBC36	At1g16890^(a)^	UBC13B	XV	17,220	Yes	Yes
UBC37	At3g24515^(c)^		XVI	45,080	No	nd

**Fig. 2:**
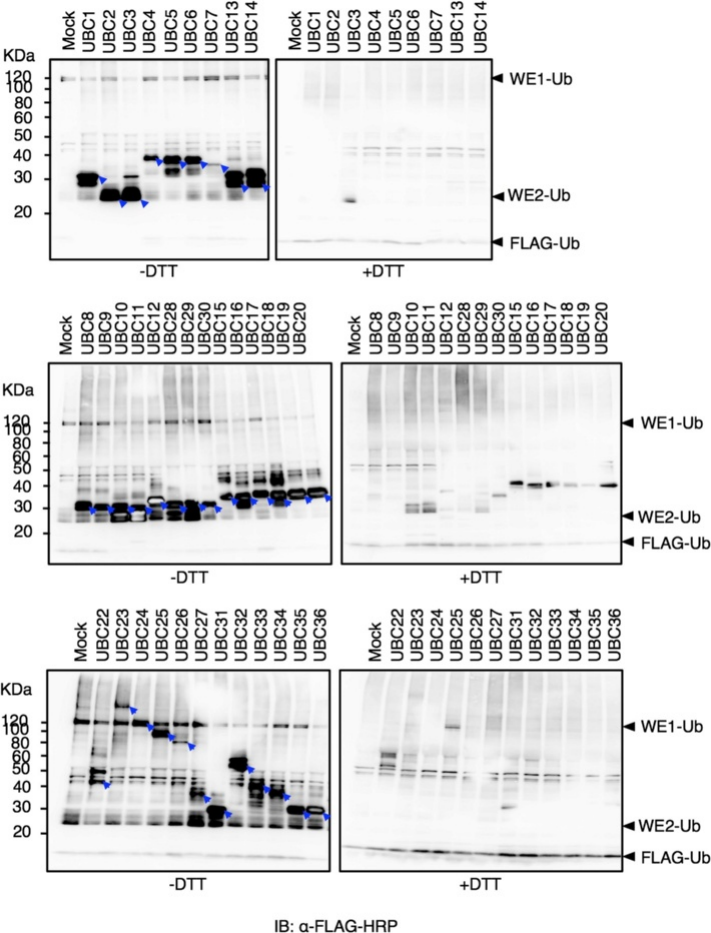
Thioester assay of 35 Arabidopsis E2s. The crude proteins for each of the 35 bio-E2s were incubated with FLAG-Ub for 5 min at 37 °C and treated with DTT or 8 M urea (-DTT). Immunoblot analysis against FLAG-Ub using anti-FLAG-HRP antibodies shows the presence of DTT-sensitive Ub conjugation activity for all E2s tested. Arrows show the expected E2-Ub adduct for each E2 in the absence of DTT. The side arrows show the free FLAG-Ub and expected FLAG-Ub adducts with WE2 and WE1 as arranged from bottom to top

The UBC names and subfamilies used here are based on the nomenclature and classification of Arabidopsis E2s described previously [13]. ‘M. wt. (Da)’ indicates the expected molecular weight of the expressed proteins according to the RAFL database or TAIR v10. Abbreviations: yes, detected; No, not detected; nd, proteins not assayed for activity. ‘Thioester formation’ indicates whether DTT-sensitive Ub adducts for E2s were observed (Fig. 2). ^a^cDNA from RAFL. ^b^ORF was amplified from a commercially available Arabidopsis cDNA library (Stratagene). ^c^ORF was amplified by nested PCR from cDNA of 2-week-old plants treated with 100 μM ABA

### All expressed E2s catalysed DTT-sensitive Ub conjugation *in vitro*

After 35 Arabidopsis E2s were expressed using the wheat germ cell-free system, it was important to check whether these expressed proteins were functionally active *in vitro*, in particular the six E2s whose expression had not previously been reported. E2s activity are determined either through their ability to form a thioester linkage with Ub in a ‘thioester assay’, which is independent of an E3, or through their requirement in the ubiquitination activity of specific RING E3s. Because of the uncertainty in E2-E3 specificity and the large number of RING E3s in the Arabidopsis genome, we preferred to use the E3-independent thioester assay for all expressed E2s. In this type of assay, the reactions are terminated under reducing conditions (SDS sample buffer with DTT) or under non-reducing conditions (SDS sample buffer with 8 M urea). In contrast to the 8 M urea treatment, the DTT treatment cleaves the thioester linkage between the E2 active site cysteine and the carboxyl terminus of Ub [24].

To evaluate the possibility of conducting thioester assays using the E2-containing wheat germ extract, we tested N-bio-UBC1 as a representative E2 for its ability to form thioester linkage with Ub in the presence or absence of FLAG-tagged Ub (FLAG-Ub) and/or rabbit E1 (Additional file 1). Immunoblotting analysis using anti-FLAG-HRP (Additional file 1A), revealed DTT-sensitive Ub conjugation regardless of the addition of E1 (as shown by the two bottom arrows), which suggests activity of the WE1. This figure also reveals another DTT-sensitive signal equivalent to that of E1-Ub was detected by an anti-FLAG antibody in the absence of E1 (as shown in the upper arrow on the 2^nd^ lane), which also refers to WE1 activity. Immunoblot analysis with streptavidin-HRP detected the unmodified bio-UBC1 (Additional file 1B, as shown by the lower arrow) and revealed an additional DTT-sensitive band shifted in size equivalent to single Ub adduct (as shown by the top arrow). The band shift was also detected regardless of the addition of FLAG-Ub suggesting the presence of endogenous wheat germ Ub. Taken together, these results confirm the activity of wheat germ endogenous E1 and the presence of Ub in the wheat germ extract, consistent with a previous report [25].

Accordingly, we tested the activity of 35 N-bio-E2s by a wheat germ-based thioester assay, relying on the activity of endogenous E1. Remarkably, all 35 E2s expressed were able to catalyse DTT-sensitive Ub conjugation in *in vitro* assays based on the wheat germ extract after blotting against FLAG-Ub (Fig. 2, summarized in Table 1). This included six E2s that have never been expressed before (UBC12, UBC23, UBC24, UBC25, UBC31, and UBC33), and other E2s that were expressed in previous studies but showed no activity (UBC16, UBC17, UBC18, UBC20, UBC26), and E2s that activated certain E3s but were not successful in thioester linkage formation with Ub (UBC3, UBC5, UBC6, UBC29, UBC30, UBC22, and UBC34). Some E2s including UBC15, UBC16, UBC17, UBC18 and UBC22 appeared as two bands on immunoblot analysis. Background of probable wheat germ endogenous E2s (WE2s) was detected at about 25–30 KDa, but fortunately, those signals were weak enough to allow the activity of recombinant E2s to be distinguished (Fig. 2). Since wheat germ extract may contain active E3s [26], we were unable to determine whether the activity of these E2s depended on the presence of a specific E3 or other protein(s), such as an activator.

### A total of 204 Arabidopsis RING proteins were expressed using the wheat germ cell-free system

**Fig. 3:**
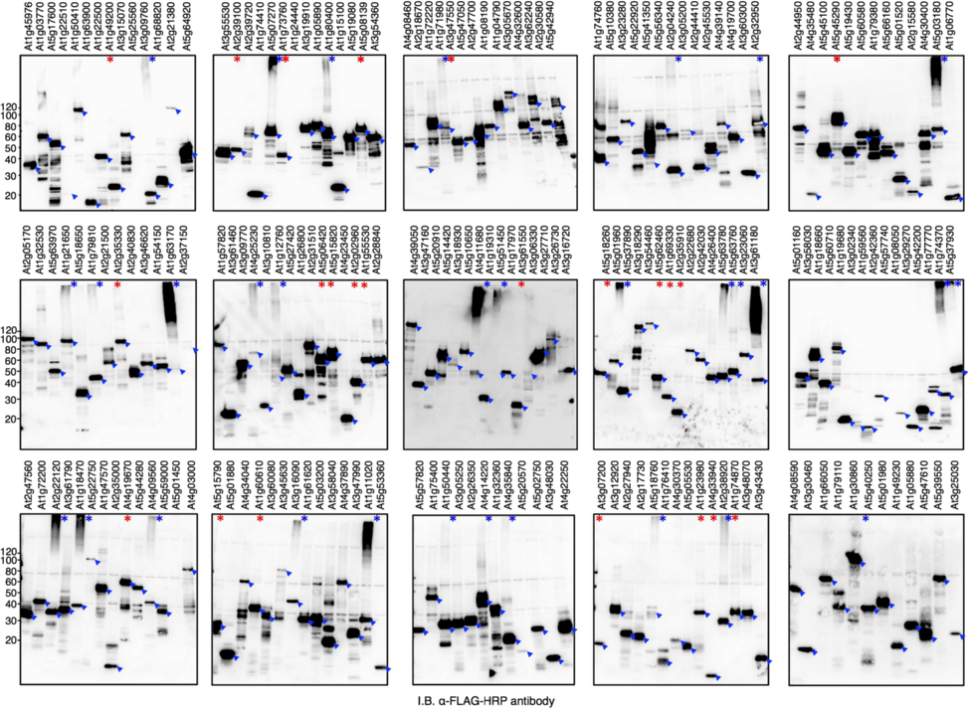
Immunoblot analysis of the N-terminal FLAG-tagged RING protein library expressed by the wheat germ cell-free system. For analysis, 2 μL of crude recombinant RING proteins with N-terminus FLAG tag was loaded onto SDS-PAGE and detected by anti-FLAG-HRP antibody. A total of 204 out of 208 RING proteins analysed were detected. Arrows show the expected signal for each RING protein. Blue asterisks refer to proteins with high molecular smears, while red asterisks refer to RING proteins did not show high molecular smears and were subsequently used in the *in vitro* ubiquitination analysis (Fig. 5, Fig. 6)

The Arabidopsis genome is predicted to encode more than 470 RING domain-containing proteins [14]. To construct a protein library of Arabidopsis RING proteins, we collected 274 cDNA clones from the RAFL library [21] according to the annotated RING proteins [14] and annotated genes in RAFL database [21]. We prepared transcription templates with an N-terminal FLAG-tag sequence using the ‘split-primer’ PCR method (Additional file 2). We were able to construct transcription templates for 208 RING clones (about 75 % of the clones collected) (Additional file 3). Following expression using the bilayer mode of the wheat germ cell-free system, expression was confirmed for 204 of the 208 RING protein-encoding mRNAs by immunoblot analysis (Fig. 3). Fifteen RNAs were expressed at relatively low levels. We compared the sizes of the expressed proteins against the expected molecular weights, as recorded in the RAFL database. We note that not all cDNA clones from RAFL matched the representative gene model in TAIR v10. Therefore, we mainly used the RAFL information to make comparisons since it was the source of the cDNAs used in the synthesis of our RING protein library. Accordingly, all but seven of the 204 expressed proteins had molecular weights that match those predicted (+/- 20 KDa). These seven proteins were > 20 KDa smaller than their expected RAFL sizes and were considered to be truncated (Additional file 3). Interestingly, upon detection of expressed RING proteins, we noticed a group of 31 proteins with anti-FLAG high molecular smears or with immunoreactivity at very high molecular masses near the top of the resolving gel (Fig. 3, with blue asterisks; Table 2). Because RING proteins are predicted to function as Ub E3 ligases and the wheat germ extract contains endogenous E1, E2, and Ub, we hypothesized that these smears and high molecular mass forms result from Ub ligase activity.

**Table 2:** Representative Arabidopsis RING proteins used in this study, their expression in a wheat germ cell-free system and their ubiquitination activities

AGI loci	Other names	RING type	M. wt. (Da)	High molecular smear	Activity with UBC8/10	Activity with other UBCs	Comments
At1g22500	ATL15	H2	42,226	No^(a)^	Yes^(c)^	UBC11, UBC28 and UBC29^(e)^	
At1g49200		H2	24,790	Yes^(b)^	nd	nd	
At3g09760		v	23,097	Yes^(a)^	Yes^(c)^	UBC11^(e)^	
At2g39100		HCa	31,376	No^(a)^	Yes^(d)^	nd	
At1g74410		H2	12,293	No^(a)^	No^(c)^, Yes^(d)^	UBC8^(f)^	
At5g07270	XBAT33	HCa	56,010	Yes^(a)^	Yes^(c)^	UBC11, UBC28^(e)^	
At1g73760		H2	40,616	No^(a)^	Yes^(d)^	nd	
At1g80400		H2	44,551	Yes^(a)^	nd	nd	
At1g71980		H2	46,218	Yes^(a)^	nd	nd	
At1g15100	RHA2a	H2	16,983	No^(a)^	Yes^(c)^	UBC11, UBC28 and UBC29^(e)^	
At5g08139		H2	60,426	Yes^(b)^	nd	nd	
At2g18670		H2	21,067	No^(a)^	No^(c)^	nd	Low expression
At3g47550		v	31,784	Yes^(b)^	nd	nd	
At2g47700	RFI2	H2	35,729	No^(a)^	Yes^(c)^	UBC11, UBC28 and UBC29^(e)^	
At1g74760		H2	29,318	No^(a)^	No^(c)^	nd	
At3g23280	XBAT35	HCa	50,055	No^(a)^	Yes^(c)^	UBC11, UBC28^(e)^	
At2g04240	XERICO	H2	17,928	Yes^(a)^	nd	nd	
At5g22920	RZPF34	H2	33,549	No^(a)^	No^(c)^	nd	
At3g05200	ATL6	H2	42,561	No^(a)^	Yes^(c)^	UBC11, UBC28 and UBC29^(e)^	Low expression
At4g39140		H2	47,548	No^(a)^	No^(c)^	nd	
At2g32950	COP1/DET340/EMB168/FUS1	HCa	76,188	Yes^(a)^	nd	nd	
At2g44950	HUB1/RDO4	HCa	59,491	No^(a)^	Yes^(d)^	UBC1, UBC2 and UBC8^(f)^	
At5g45290		H2	60,928	No^(a)^	Yes^(d)^	nd	
At5g01520	AIRP2	HCa	28,050	No^(a)^	No^(c)^	nd	
At2g15580		H2	23,182	No^(a)^	No^(c)^, Yes^(d)^	UBC8, UBC31^(f)^	
At5g03180		v	52,475	Yes^(a)^	nd	nd	
At1g21650	SECA2		177,644	Yes^(a)^	nd	nd	Truncated
At1g79810	PEX2/TED3	HCa	38,175	Yes^(a)^	nd	nd	
At2g35330		HCa	79,194	Yes^(a)^	Yes^(d)^	nd	
At1g63170		H2	42,677	Yes^(a)^	nd	nd	
At3g09770	AIRP3/LOG2	HCa	42,848	No^(a)^	Yes^(c)^	UBC11, UBC28 and UBC29^(e)^	
At4g25230	RIN2	H2	66,537	Yes^(a)^	nd	nd	Low expression
At1g12760		H2	40,453	Yes^(a)^	Yes^(c)^	UBC11, UBC28 and UBC29^(e)^	Low expression
At5g06420		HCa	42,460	No^(a)^	Yes^(d)^	nd	
At5g15820		H2	38,560	Yes^(b)^	nd	nd	
At4g23450	AIRP1	H2	21,143	No^(a)^	Yes^(c)^	UBC11, UBC28 and UBC29^(e)^	
At2g02960		v	29,591	Yes^(b)^	nd	nd	
At1g55530		H2	38,963	Yes^(b)^	nd	nd	
At2g28840	XBAT31	HCa	46,391	No^(a)^	No^(c)^	nd	
At3g47160		HCa	28,524	No^(a)^	Yes^(c)^	UBC11^(e)^	
At5g20910	AIP2	H2	34,807	No^(a)^	Yes^(c)^	UBC11, UBC28, UBC29 and UBC30^(e)^	
At5g14420	RGLG2	HCa	51,578	No^(a)^	Yes^(c)^	UBC11, UBC28, UBC29 and UBC30^(e)^	
At4g11680		H2	47,218	Yes^(a)^	Yes^(c)^	UBC11, UBC28, UBC29, UBC35 and UBC36^(e)^	
At5g51450	RIN3	H2	65,087	Yes^(a)^	nd	nd	Low expression
At3g61550		H2	23,224	No^(a)^	Yes^(d)^	nd	
At3g06330		v	46,711	No^(a)^	Yes^(c)^	UBC11, UBC28 and UBC29^(e)^	Low expression
At3g16720	ATL2	H2	34,052	No^(a)^	No^(c)^, Yes^(d)^	UBC8, UBC31^(f)^	
At5g18260		H2	35,673	No^(a)^	Yes^(d)^	nd	
At5g01960		HCa	46,018	Yes^(a)^	nd	nd	
At5g62460		v	33,660	Yes^(b)^	nd	nd	
At1g69330		HCa	30,423	No^(a)^	Yes^(d)^	nd	
At2g35910		H2	19,725	No^(a)^	Yes^(d)^	nd	
At2g22680	WAVH1	H2	74,401	No^(a)^	Yes^(c)^	UBC11, UBC28 and UBC29^(e)^	
At5g63780	SHA1	H2	39,838	Yes^(a)^	nd	nd	
At5g63760	ARI15	HCb	57,610	Yes^(a)^	nd	nd	
At3g61180		H2	40,754	Yes^(a)^	nd	nd	
At1g74370		HCa	29,400	Yes^(a)^	Yes^(c)^	UBC11, UBC28, UBC29, UBC35 and UBC36^(e)^	
At5g37930		HCa	38,924	Yes^(a)^	nd	nd	
At2g22120		v	28,253	Yes^(a)^	nd	nd	
At1g18470		HCa	47,408	Yes^(a)^	nd	nd	
At4g19670		HCb	60,343	No^(a)^	Yes^(d)^	nd	
At4g09560		H2	48,030	Yes^(a)^	nd	nd	
At5g15790		H2	26,352	No^(a)^	Yes^(d)^	nd	
At1g60610		HCa	38,334	No^(a)^	No^(c)^, Yes^(d)^	nd	
At3g16090	HRD1a	H2	56,037	Yes^(a)^	nd	nd	
At1g11020		v	31,326	Yes^(a)^	nd	nd	
At1g50440		H2	28,785	Yes^(a)^	Yes^(c)^	nd	
At4g14220	RHF1a	H2	41,035	Yes^(a)^	Yes^(c)^	nd	
At4g35840		H2	29,169	Yes^(a)^	nd	nd	
At3g07200		HCa	20,090	Yes^(b)^	nd	nd	
At1g23980		H2	40,765	No^(a)^	Yes^(d)^	nd	
At4g33940		HCa	73,413	No^(a)^	Yes^(d)^	nd	Truncated
At2g38920		HCa	35,490	Yes^(a)^	nd	nd	
At1g74870		C2	33,404	No^(a)^	Yes^(d)^	nd	
At3g48070		C2	35,234	No^(a)^	Yes^(c)^	UBC11^(e)^	
At1g30860		HCa	84,259	Yes^(a)^	nd	nd	

The table shows the expression and ubiquitination activities of representative Arabidopsis RING proteins used in this study (See additional file 3 for a summary of all RING ORFs used in this study). ‘M. wt. (Da)’ indicates the expected molecular weight of the expressed proteins according to the RAFL database. ‘High molecular smear’ indicates the detection of a smear: ^a^when RING protein expression was detected by immunoblot analysis or ^b^when analysed in ubiquitination assays without the addition of E1 or E2. ‘Activity with UBC8/10’ indicates the E3 ligase activity of selected RING proteins tested with ^c^AtUBC8 [14] or ^d^AtUBC10 in this study. ‘Activity with other E2s’ indicates the E3 ligase activity of selected RING proteins tested along with various E2s in ^e^previous study [13] or ^f^in this study. ‘Truncated’ refers to proteins that were more than 20 KDa less than their expected size. ‘Low expression’ refers to proteins expressed at relatively low levels. Abbreviations: yes, detected; No, not detected; nd, not tested

### RING proteins catalyse ubiquitination activity using WE1 and WE2

**Fig. 4:**
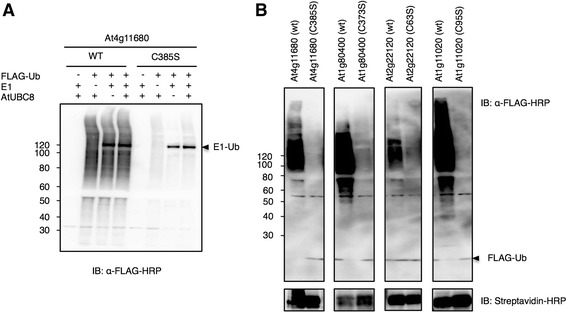
Wheat germ-based *in vitro* ubiquitination analysis of RING proteins showed high molecular smears. **a** At4g11680 and its corresponding RING mutant were expressed with biotin tag and analysed as a representative protein in the presence or absence of FLAG-Ub, E1, and/or AtUBC8 without tag for the ubiquitination activity. Replacement of the third metal ligand, Cys to Ser, caused reduced smear upon blotting against FLAG-Ub with anti-FLAG-HRP antibody. Absence of the E1 or AtUBC8 from the reaction did not abolish the high molecular smear. **b** Three other RING proteins At1g80400, At2g22120, and At1g11020, and their RING mutants together with At4g11680, were analysed in the presence of FLAG-Ub. The four proteins with RING mutants showed significantly reduced activity upon blotting with anti-FLAG-HRP antibody, whereas the proteins with intact RING domains showed activity without the addition of E1 or E2. The side arrow refers to the free FLAG-Ub that migrated to the bottom of the gel

To verify whether the smears and high molecular mass forms that appeared after the expression of some RING proteins resulted from RING activity in the extract, we introduced a point mutation at the codon for the third metal ligand residue required for maintaining the RING domain structure and function (substituting a serine codon for a cysteine) [14]. We selected At4g11680 as a representative RING protein for this experiment, because it produced a readily detectable high molecular smear after expression (Fig. 3). An *in vitro* ubiquitination assay using wild-type N-bio-At4g11680 (wt) and its corresponding RING mutant N-bio-At4g11680 (C385S) was performed. As shown in Fig. 4a, At4g11680 (wt) promoted production of a high molecular weight smear with or without added E1 or E2 (AtUBC8). In contrast, the RING mutant of At4g11680 (C385S) showed a significantly diminished ability to promote the production of a high molecular weight smear. This result indicates that RING protein activity is required for production of a Ub smear and for these proteins is independent of Arabidopsis E1 and E2, likely utilizing WE1 and WE2. To further test this hypothesis, we selected three other RING proteins that also showed high molecular smears when expressed. These were At1g80400, At2g22120, and At1g11020. These proteins as well as At4g11680 were expressed with biotin tags. Similarly, a point mutation was introduced at the codon for the third metal ligand residue of each protein, and both forms were tested using *in vitro* ubiquitination assays. The ability of the RING mutants to promote ubiquitination was drastically diminished in comparison to the activity of their corresponding wild-type proteins (Fig. 4b). These data demonstrate that the expressed RING proteins have functional activity using WE1 and WE2. In addition, the accumulation of ubiquitinated proteins when expressed in the wheat germ cell-free system also suggests the presence of endogenous Ub and reduced or inactivity of the wheat germ 26S proteasome, which is also consistent with previous report [25].

### RING proteins exhibit ubiquitination activity with AtUBC10

**Fig. 5:**
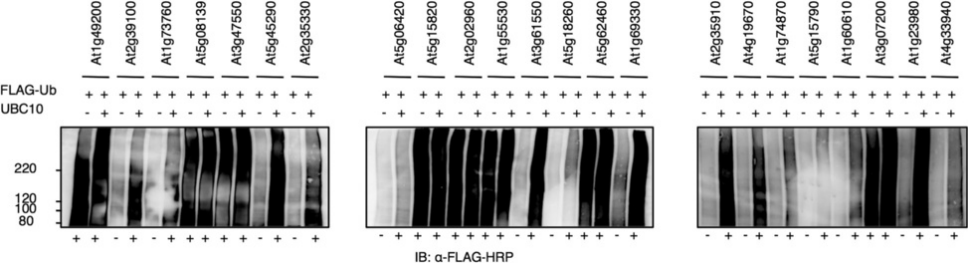
Wheat germ-based *in vitro* ubiquitination assays of various types of RING proteins. 23 FLAG-tagged RING proteins of various RING types were mixed with FLAG-Ub in the presence or absence of N-bio-UBC10 as indicated by plus (+) or minus (–) above each lane. E3 activity was determined by the presence or absence of a smear when FLAG-Ub was detected by anti-FLAG-HRP antibody. This is indicated by plus (+) or minus (–), respectively, below each lane respectively

While 31 RING proteins had clear high molecular smears when expressed in the wheat germ cell-free system indicating activity, the majority of the other RING proteins expressed did not exhibit activity. To test the functional activity of these RING proteins we conducted *in vitro* ubiquitination assays for 23 RING proteins with the addition of FLAG-Ub and N-bio-UBC10 to increase the sensitivity of the assay and to see whether Arabidopsis E2 is essential. The 23 RING proteins included various types of RING proteins (Table 2, Additional file 3). Some RING proteins showed polyubiquitination activity only after the addition of UBC10, suggesting that these RING proteins require this Arabidopsis E2 (Fig. 5) or that this E2 type is not present in wheat germ. In contrast, other RING proteins showed polyubiquitination activity in the absence of UBC10, suggesting that these RING proteins can exhibit weak activity using WE2, which can be detected clearly after adding FLAG-Ub.

### RING proteins exhibit ubiquitination activity with different E2 subfamilies

**Fig. 6:**
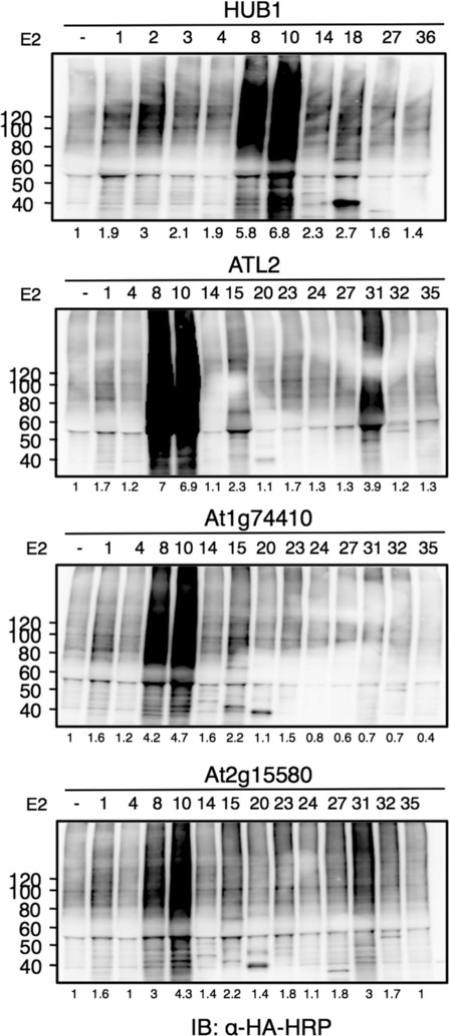
Wheat germ-based *in vitro* E2-E3 specificity screening. The activity of four FLAG-tagged RING E3s was tested in the presence of members of different subgroups of N-bio-E2s and HA-Ub. Arabidopsis E2s used in each assay are indicated above each lane according to their UBC number and minus (–) refers to the absence of E2; this condition was used as a negative control. The E2–E3 activity was visualized after SDS-PAGE by anti-HA-HRP immunoblotting. The number below each lane represents the signal intensity quantified by imageJ as normalized to the lane lacking for E2 (–)

To further test the functional activity of RING proteins, we selected three RING proteins (ATL2, At3g74410, and At3g15580) that were reported to be inactive when tested with different Arabidopsis E2s [13]. Another RING protein, which mediates monoubiquitination of histone H2B named HISTONE MONOUBIQUITINATION 1 (HUB1), was also tested. HUB1 was reported to specifically use UBC1 and UBC2 for monoubiquitination of H2B *in vitro* and *in vivo* [27]; therefore, it was of interest to see whether other E2s could promote HUB1 activity. We tested the activity of these four proteins using the wheat germ-based *in vitro* ubiquitination assays in the presence of E2s from different subgroups. All the RING proteins tested showed relatively intensive smears with two subfamily VI E2s UBC8 and UBC10 (Fig. 6) and in some cases with subfamily XIII E2 UBC31. For At2g15580, smears in the presence of UBC10 was the most pronounced, while that in the presence of UBC8 and UBC31 was slightly lower. HUB1 showed strong smears with UBC8 and UBC10, and moderate one with UBC2. ATL2 also showed relatively intensive smears with UBC31 as well as UBC8 and UBC10. Taken together, these results suggest the activity of some RING proteins that did not exhibit activity when expressed previously using *E. coli* cells, indicating the importance of the eukaryotic expression system in functional analysis of Arabidopsis proteins.

## Discussion

Complete genome sequences make it substantially easier to deduce the function of genes by identifying conserved domains in the putative proteins encoded by these genes [28]. However, definitive assignment requires experimental verification. For the genes encoding the enzymatic core of the ubiquitination pathway in Arabidopsis, a large fraction of the proteins encoded by these genes are biochemically uncharacterised. In this study, we expressed about 95 % of the Arabidopsis E2 proteome (35 out of 37 enzymes) using the wheat germ cell-free system (Fig. 1b). This protein library included all of the E2s that have been previously characterised [8, 13, 27, 29]. Members of subfamily XIV, which contain predicted transmembrane domains, were expressed without deletions. In addition, members of subfamily XI, which are exclusively large proteins, and some members of subfamily VII; UBCs 16–18, were not expressed using *E. coli* or cultured insect cells [13], but were successfully expressed using the wheat germ cell-free system. Surprisingly, all of the 35 expressed E2s showed DTT-sensitive Ub conjugation activity (Fig. 2), confirming the advantage of the eukaryotic wheat germ-based protein expression system for studying eukaryotic protein functions. Through this study, we provided the largest collection of the most important enzymes in the ubiquitination process in functional form. This advantage makes the *in vitro* analysis of various E3s possible and can improve our understanding of E2-E3 specificity in Arabidopsis.

Using the wheat germ cell-free system, we were also able to synthesize a protein library of 204 Arabidopsis RING E3s, which represents more than 40 % of the Arabidopsis RING proteome (Fig. 3). To our knowledge, this is also the largest collection of expressed RING proteins reported. The availability of full-length cDNA libraries like RAFL and a PCR-based method for preparing linear transcription templates like the ‘split-primer’ PCR enabled us to explore the products of such large number of genes *in vitro*. In comparison with the conventional cloning and expression approach, our approach bypassed the time-consuming subcloning steps into expression vectors and led to high-throughput protein expression when combined with the wheat germ cell-free system [22]. We have demonstrated the activity of 27 RING proteins as E3 ligases, 17 of them were demonstrated for the first time (Table 2).

It is important to note that the 204 genes were amplified under similar conditions, and transcribed and translated under the same conditions for the high-throughput synthesis of the protein library. Future modifications to the PCR conditions according to the nature of each template cDNA may yield more transcription templates. Furthermore, in our study we could collect from the RAFL cDNA library 274 RING clones out of about 470 annotated RING genes. The addition of the remaining RING cDNAs, from sources other than the RAFL cDNA library, may improve the coverage of our protein library, and would improve any future analyses.

The thioester and *in vitro* ubiquitination assays normally require protein purification from either recombinant expression or from its native source and the addition of commercially available components such as Ub and E1. Accordingly, large scale *in vitro* analysis could be laborious and costly. E2s such as AtUBC22 and AtUBC35, and RING E3s such as CIP8 were reported to catalyse ubiquitination when analysed using wheat germ crude extracts without exogenous E1 [25]. Therefore, we tested the activity of 35 E2s and several E3s using *in vitro* assays based on their crude extract without purification and without addition of E1. Blotting against FLAG-Ub in thioester assays of E2s like UBC15, UBC16, UBC17, UBC18 and UBC22 showed two bands (Fig. 2). In case of UBC22, previous reports clearly showed its unique capability to conjugate with one or more Ub molecules (13, 24). While in case of other E2s, further investigations are required to examine whether they may have similar capability or this extra band appeared because of unspecific Ub addition on E2, its tag or Ub tag.

The detection of ubiquitinated species after RING protein expression (Fig. 3, Fig. 4) also suggests the stability of ubiquitinated proteins in the wheat germ extract. This finding is consistent with a previous study [25], and may suggest that 26S proteasomal activity is absent in the wheat germ extract. Verification of proteasomal activity has become possible using fluorescent reporters [30]. All the 27 E3s tested in our study showed activity with UBC10 and/or UBC8, which are related to the human UbcH5 family. These E2s are abundantly expressed in almost all plant organs [13] and considered as promiscuous E2s [8]. SO, it is not surprising that they can function with most E3s. Even HUB1 E3 which has been shown to specifically use UBC1 and UBC2 for monoubiquitination of H2B [27], showed strong activity with UBC8 and UBC10 in our study. That suggests that UBC8 and UBC10 may perform a general ubiquitination function *in vivo* while other E2s like UBC1 and UBC2 may be involved in specific functions like histone modification. The E2-E3 specificity analysis performed in this study could offer a potential dataset, which can be helpful for future *in vivo* analysis.

The use of wheat germ-based protein libraries such as those described here, is not limited to the analysis of gene products but can be used as a platform for several perspective studies. Our RING protein library can be used to screen for protein-protein interactions and contribute to the discovery of novel RING E3-substrate relationship in Arabidopsis plants. Some RING E3s ligases showed significant interaction signals when screened with Arabidopsis key regulatory proteins using AlphaScreen protein-protein interaction screening technology (unpublished data). In the future, modifying the wheat germ extract by removing some endogenous components, such as WE1(s) and/or WE2(s), and the possible isolation of intact Arabidopsis 26S proteasomes [31], may increase our understanding of the Ub proteasome system in Arabidopsis. The ease by which the wheat germ system produces proteins with different N-terminal sequences and the absence of 26S proteasomal activity makes the system suitable for systematic analysis of the N-end rule [32]. The availability of functionally active protein libraries for E2 and RING E3s enzymes is an important step in improving our understanding of E2–E3 interactions and specificity. Expanding our system to analyse substrate ubiquitination using different E2–E3 combinations may also increase our understanding of the various cellular processes that are regulated by ubiquitination.

## Conclusion

In this study, we demonstrated the importance of using a eukaryotic and plant-related protein expression system in performing *in vitro* analysis of Arabidopsis E2s and RING E3s. Using a combination of the RAFL cDNA library, the ‘split-primer’ PCR method and the wheat germ cell-free system, we were able to express about 95 and 40 % of the Arabidopsis E2 and RING-type E3 proteomes, respectively. The functional activities of several proteins were assessed in this study for the first time. The protein libraries described here can be used to improve understanding E2-E3 specificities and as platforms for identifying new target substrates through protein-protein interaction screening.

## Methods

### Plant material

Seeds of Arabidopsis ecotype Col-0 were obtained from Riken bioresource center. The seeds were sterilized with 30 % (v/v) bleach and grown on 1 % (w/v) agar with 0.5× Murashige and Skoog medium and 3 % (w/v) Suc under continuous light.

### Cloning and construction of DNA templates for transcription

From the annotated E2s and RING proteins, we collected 29 UBC and 274 RING clones from the RIKEN Arabidopsis full-length (RAFL) cDNA library. Other five UBCs AtUBC7, AtUBC17, AtUBC21, AtUBC23, AtUBC31, and AtUBC35 were cloned into pT7Blue T-vector or pDONR221 (TakaraBio, http://www.takara-bio.com/) after PCR amplification from a commercially available Arabidopsis cDNA library (Stratagene, http://www.stratagene.com/). AtUBC12 and AtUBC37 were amplified by nested PCR from cDNA of 2-week-old Arabidopsis plants, ecotype Col-0, treated with 100 μM ABA. The amplified inserts were cloned in pDONR221 vector using the gateway cloning system (Invitrogen, Carlsbad, CA) and their sequences were confirmed. Sequences of the eight clones were compared with the predicted ORF available on TAIR v10 (http://www.arabidopsis.org). The Qiagen RNeasy plant RNA extraction kit (Qiagen, Valencia, CA) was used to isolate total RNA according to the manufacturer’s instructions. The source of cDNA for UBCs and RING clones is outlined in Table 1 and Additional file 3, respectively.

The high-throughput construction of transcription templates was performed by the “split-primer” PCR method (Fig. 1a and Additional file 2), as previously described [22]. Primer sequences employed in this study are listed in Additional file 4. The first round of PCR was performed using 100 nM of the following primers: a gene specific primer-S1, 5’-CCACCCACCACCACCAATGnnnnnnnnnnnnnnnnnnn (n denotes the sequence complementary to the first 20 bp in the coding region of the target gene), and AODA2306 or AODS based on the insert orientation. Next, a second round of PCR was carried out to add the promoter SP6, the translation enhancer sequence E01, and tag sequence at the 5’ end of the ORF using the first PCR product as template. Two sense primers were used in the PCR reaction, along with 100 nM SPu primer, and 1 nM of either deSP6E02FLAG-S1 or deSP6E02bls-S1 to produce an N-terminal FLAG-tagged or N-terminal biotin ligation site (bls)-fused transcription templates respectively. The antisense primer used was 100 nM AODA2303 or pDONR221 2^nd^A4035. Gene-specific primers for ‘split-primer’ PCR were designed according to the cDNA sequences deposited in the RAFL database [21] or TAIR v10 (http://www.arabidopsis.org).

### Mutagenesis

For RING mutational analysis, we designed primers to generate a point mutation at the 3^rd^ metal ligand residue (Additional file 5) using PrimeSTAR MAX DNA polymerase (Takara, Kyoto, Japan) and pDONR221 vector containing the RING cDNA as a PCR template. Sequence analysis was used to confirm nucleotide changes.

### Cell-free protein synthesis

*In vitro* transcription and cell-free protein synthesis were performed as described previously [33]. Transcripts were made from each DNA template using SP6 RNA polymerase. The synthetic mRNAs were then precipitated with ethanol and collected by centrifugation using a Hitachi R10H rotor. Each mRNA was washed and transferred into a translation reaction mixture. The translation reaction was performed in the bilayer mode according as previously described [22]. The translation mixture for the bottom layer consisted of 60 A_260_ units of the wheat germ extract (Cell-Free Sciences, http://www.cfsciences.com), and 2 μg creatine kinase (Roche Diagnostics K.K., http://www.roche-diagnostics.jp) in 25 μL SUB-AMIX (Cell-Free Sciences). The SUB-AMIX contained (final concentrations) 30 mM Hepes/KOH at pH 8.0, 1.2 mM ATP, 0.25 mM GTP, 16 mM creatine phosphate, 4 mM DTT, 0.4 mM spermidine, 0.3 mM each of the 20 amino acids, 2.7 mM magnesium acetate, and 100 mM potassium acetate. A total of 125 μL SUB-AMIX was placed first as the upper layer, and the bottom layer was then pipetted gently underneath. For biotin labelling, 1 μL of crude biotin ligase (BirA) produced by the wheat cell-free expression system was added to the bottom layer, and 0.5 μM (final concentration) of D-biotin (Nacalai Tesque, Inc., http://www.nacalai.co.jp) was added to both layers, as described previously [34]. After incubation at 16 °C for ~20 h, each protein was separated into aliquots of 10 μl, frozen by liquid nitrogen and stored at -80 °C. The aliquots were used for the expression analysis and functional characterisation.

### Immunoblot analysis

The expression of recombinant FLAG-tagged RING and biotinylated UBC proteins was detected by immunoblot analysis. A sample of 2–6 μL of recombinant proteins was mixed with 3× SDS sample buffer then boiled for 5 min. The denatured proteins were separated on 5–20 % SDS-PAGE and transferred to PVDF membrane using the iBlot dry blotting system (Invitrogen, http://www.lifetechnologies.com) or EzFastBlot (ATTO, http://www.atto.co.jp). Immunoblot analysis was carried out with monoclonal anti-FLAG M2-peroxidase (HRP) antibody (Sigma) or conjugated streptavidin-HRP (Invitrogen) and detected by using Immobilon Western Chemiluminescent HRP Substrate (Millipore, http://www.millipore.com), according to the manufacturer’s procedure. Finally, the signals were visualized using ImageQuant LAS 4000 mini (GE Healthcare, http://www.gelifesciences.com). ‬‬‬‬‬‬‬‬‬‬‬‬‬‬‬‬‬‬‬‬‬‬‬‬‬‬‬‬‬‬‬‬‬‬‬‬‬‬‬‬‬‬‬‬‬‬‬‬‬‬‬‬‬‬‬‬‬‬‬‬‬‬‬‬‬‬‬‬‬‬‬‬‬‬

### Thioester assay

Thioester assays were performed as previously described [13], with slight modifications because the assays in this study were based on wheat germ extract and it’s E1 (WE1). In a total reaction volume of 25 μL, we mixed 20 mM Tris-HCl, pH 7.4, 5 mM MgCl_2_, 3 mM ATP, 1 mg/ml BSA, 400 nM human recombinant FLAG-Ub (Boston Biochem, http://www.bostonbiochem.com), 3 μL recombinant E2s. The reaction mixtures were incubated for 5 min at 37 °C and the reaction was terminated by the addition of 2× SDS sample buffer with DTT or 8 M urea sample buffer without boiling. SDS-PAGE was performed at 4 °C followed by immunoblotting using anti-FLAG-HRP. A 10-μL sample was used for SDS-PAGE loading with the exception of UBC22, UBC23, UBC24, UBC25, and UBC26, for which 15 μL was used.

### *In vitro* ubiquitination assay

The wheat germ-based ubiquitination assays of RING proteins were carried out as previously described [25]. The assays were performed in a 10 μL reaction mixture containing 20 mM Tris-HCl pH 7.5, 0.2 mM DTT, 5 mM MgCl_2_, 10 μM zinc acetate, 3 mM ATP, 1 mg/mL BSA, 400 nM human recombinant FLAG-Ub or HA-Ub (Boston Biochem, http://www.bostonbiochem.com), 1 μL recombinant E2, and 1 μL recombinant RING protein at 37 °C for 3 h. The reactions were terminated by the addition of 5 μL 3× SDS sample buffer and boiling for 5 min, and were then analysed on 5–20 % SDS-PAGE followed by immunoblotting using anti-FLAG-HRP (Sigma) or anti-HA-HRP (clone 3 F10) antibodies (Roche Life Science).

### Availability of supporting data

All the supporting data are included as additional files.

## Additional files

Additional file 1:
**Wheat germ-based thioester assay of UBC1.** Wheat germ-based thioester assay of UBC1. Bio-UBC1 crude protein was analyzed as a representative E2 in the presence or absence of FLAG-Ub and/or E1. The reactions incubated for 2 h at 37 °C and treated with DTT or 8 M urea (-DTT) (a) Immunoblot analysis with anti-FLAG-HRP antibodies show the presence of DTT-sensitive Ub conjugation activity for UBC1 regardless the addition of E1 (as referred by the two bottom arrows). (b) Immunoblot analysis with streptavidin-HRP detected the bio-UBC1 (as referred by the lower arrow) and showed DTT-sensitive band shift equivalent to single Ub adduct (as referred by the top arrow). (PPTX 198 kb) Additional file 2:
**Flow chart of the wheat germ-based procedure for the production of Arabidopsis RING protein library.** The first step involves the high-throughput preparation of DNA templates for transcription using 2 step “split-primer” PCR, followed by *in vitro* transcription using phage-coded SP6 RNA polymerase, and finally translation using wheat germ cell-free system. All the steps were carried out in 96-well microtiter plates. (PPTX 55 kb) Additional file 3:
**Summary of Arabidopsis RING clones used in this study.** We demonstrate their expression and their ubiquitination activities. Summary of Arabidopsis RING proteins used in this study, their expression in a wheat germ cell-free system and their ubiquitination activities. ‘M. wt. (Da)’ indicates the expected molecular weight of the expressed proteins according to RAFL database. ‘High molecular smear’ indicates the detection of smear: ^a^when RING protein expression was detected by immunoblot analysis or ^b^when analysed in ubiquitination assays without the addition of E1 or E2. ‘Activity with UBC8/10’ indicates the E3 ligase activity of selected RING proteins tested with ^c^AtUBC8 [14] or ^d^AtUBC10 in this study. ‘Activity with other E2s’ indicates the E3 ligase activity of selected RING proteins tested with ^e^various E2s [13] or ^f^in this study. ‘Truncated’ refers to protein that migrated more than 20 KDa less than the expected size. ‘Low expression’ refers to proteins expressed at relatively low levels. Abbreviations: yes, detected; No, not detected; nd, not tested. (XLSX 69 kb) Additional file 4:
**List of primers employed for the construction of transcription templates for Arabidopsis E2s and RING protein.** (XLSX 42 kb) Additional file 5:
**List of primers employed for RING domain mutagenesis.** (XLSX 34 kb)

## Abbreviations

RAFL: RIKEN Arabidopsis full-length cDNA library
Ub: Ubiquitin
E1: Ubiquitin-activating enzyme
E2: Ubiquitin-conjugating enzyme
E3: Ubiquitin-ligase enzyme
DTT: Dithiothreitol
HECT: Homology of the E6-AP C-terminus
RING: Really interesting new gene
UEV: Ubiquitin enzyme variants
UBL: Ubiquitin-like proteins
CRL: Cullin-RING ligase
N-bio-: N-terminus biotinylated
WE1: Wheat germ E1
WE2: Wheat germ E2
Bls: Biotin ligase site

## References

[1] Hershko A, Ciechanover A (1998). The ubiquitin system. Annu Rev Biochem.

[2] Smalle J, Vierstra RD (2004). The ubiquitin 26S proteasome proteolytic pathway. Annu Rev Plant Physiol Plant Mol Biol.

[3] Vierstra RD (2012). The expanding universe of ubiquitin and ubiquitin-like modifiers. Plant Physiol.

[4] Moon J, Parry G, Estelle M (2004). The ubiquitin-proteasome pathway and plant development. Plant Cell.

[5] Dreher K, Callis J (2007). Ubiquitin, hormones and biotic stress in plants. Annu Bot.

[6] Vierstra RD (2009). The ubiquitin/26S proteasome system at the nexus of plant biology. Nat Rev Mol Cell Biol.

[7] Stone SL. The role of ubiquitin and the 26S proteasome in plant abiotic stress signaling. Front. Plant Sci. 2014. doi:10.3389/fpls_2014_00135. 10.3389/fpls.2014.00135 PMC3997020 24795732

[8] Callis J (2014). The ubiquitination machinery of the ubiquitin system. The Arabidopsis Book.

[9] Hochstrasser M (1996). Ubiquitin-dependent protein degradation. Annu Rev Genet.

[10] Muckhopadhyay D, Riezman H (2007). Proteasome-independent functions of ubiquitin in endocytosis and signaling. Science.

[11] Hatfield PM, Gosink MM, Carpenter TB, Vierstra RD (1997). The ubiquitin-activating enzyme (E1) gene family in *Arabidopsis thaliana*. Plant J.

[12] Bachmair A, Novatchkova M, Potuschak T, Eisenhaber F (2001). Ubiquitylation in plants: A post-genomic look at a post-translational modification. Trends Plant Sci.

[13] Kraft E, Stone SL, Ma L, Su N, Gao Y, Lau OS (2005). Genome analysis and functional characterization of the E2 and RING domain E3 ligase ubiquitination enzymes of *Arabidopsis thaliana*. Plant Physiol.

[14] Stone SL, Hauksdóttir H, Troy A, Herschleb J, Kraft E, Callis J (2005). Functional analysis of the RING-type ubiquitin ligase family of Arabidopsis. Plant Physiol.

[15] Hua Z, Vierstra RD (2011). The cullin-RING ubiquitin-protein ligases. Annu Rev Plant Biol.

[16] Deshaies RJ, Joazeiro CAP (2009). RING Domain E3 Ubiquitin Ligases. Annu Rev Biochem.

[17] Lovering R, Hanson IM, Borden KL, Martin S, O’Reilly NJ, Evan GI (1993). Identification and preliminary characterization of a protein motif related to the zinc finger. Proc Natl Acad Sci U S A.

[18] Capili AD, Schlutz DC, Rausher IF, Borden KL (2001). Solution structure of the PHD domain from the KAP-1 corepressor: structural determinants of PHD, RING and LIM zinc binding domains. EMBO J.

[19] Carlson ED, Gan R, Hodgman E, Jewett MC (2012). Cell-free synthesis: Applications come of age. Biotech Adv.

[20] Endo Y, Sawasaki T (2006). Cell-free expression systems for eukaryotic protein production. Curr Opin Biotech.

[21] Seki M, Narusaka M, Kamiya A, Ishida J, Satou M, Sakurai T (2002). Functional annotation of a full-length Arabidopsis cDNA collection. Science.

[22] Sawasaki T, Ogasawara T, Morishita R, Endo Y. A cell-free protein synthesis system for high-throughput proteomics. Proc Natl Acad Sci USA. 2002;99:14652–7. 10.1073/pnas.232580399 PMC137474 12409616

[23] Zhao Q, Tian M, Li Q, Cui F, Liu L, Yin B, et al. A plant-specific *in vitro* ubiquitination analysis system. Plant J. 2013;74:524–33. 10.1111/tpj.12127 23350615

[24] Hauser P, Hofmann F. High-throughput assay to monitor formation of the E2-ubiquitin thioester intermediate. Methods in Enzymology. 2005. doi:10.1016/S0076-6879(05)98009-9 10.1016/S0076-6879(05)98009-9 16275322

[25] Takahashi H, Nozawa A, Seki M, Shinozaki K, Endo Y, Sawasaki T (2009). A simple and high-sensitivity method for analysis of ubiquitination and polyubiquitination based on wheat cell-free protein synthesis. BMC Plant Biol.

[26] Girod PA, Vierstra RD (1993). A major ubiquitin conjugation system in wheat-germ extracts involves a 15-kDa ubiquitin-conjugating enzyme (E2) homologous to the yeast UBC4/UBC5 gene-products. J Biol Chem.

[27] Cao Y, Dai Y, Cui S, Ma L (2008). Histone H2B monoubiquitination in the chromatin of flowering locus C regulates flowering time in Arabidopsis. Plant Cell.

[28] Eric SL, Lauren ML, Bruce B, Chad N, Michael CZ, Jennifer B, et al. Initial sequencing and analysis of the human genome. Nature. 2001;409:860–921. 10.1038/35057062 11237011

[29] Van Nocker S, Walker JM, Vierstra RD (1996). The Arabidopsis thaliana UBC7/13/14 gene encode a family of multiubiquitinchain-forming E2 enzymes. J Biol Chem.

[30] Dantuma NP, Lindsten K, Glas R, Jellne M, Masucci MG (2000). Short-lived green fluorescent proteins for quantifying ubiquitin/proteasome-dependent proteolysis in living cells. ‬Nat. Biotechnol.

[31] Book AJ, Gladman NP, Lee SS, Scalf M, Smith LM, Vierstra RD (2010). Affinity purification of the Arabidopsis 26S proteasome reveals a diverse array of plant proteolytic complexes. J Biol Chem.

[32] Takai K, Sawasaki T, Endo Y (2010). Practical cell-free protein synthesis system using purified wheat embryos. Nat Protoc.

[33] Sawasaki T, Gouda MD, Kawasaki T, Tsuboi T, Tozawa Y, Takai K (2005). The wheat germ cell-free expression system: methods for highthroughput materialization of genetic information. Methods Mol Biol.

[34] Sawasaki T, Kamura N, Matsunaga S, Saeki M, Tsuchimochi M, Morishita R (2008). Arabidopsis HY5 protein functions as a DNA-binding tag for purification and functional immobilization of proteins on agarose/DNA microplate. FEBS Lett.

