# Supplementary figures and images for: Wheat germ-based protein libraries for the functional characterisation of the Arabidopsis E2 ubiquitin conjugating enzymes and the RING-type E3 ubiquitin ligase enzymes
Source: BMC Plant Biol. 2015 Nov 10;15:275. doi: 10.1186/s12870-015-0660-9 (PMC4641371; doi:10.1186/s12870-015-0660-9)

## Slide 1
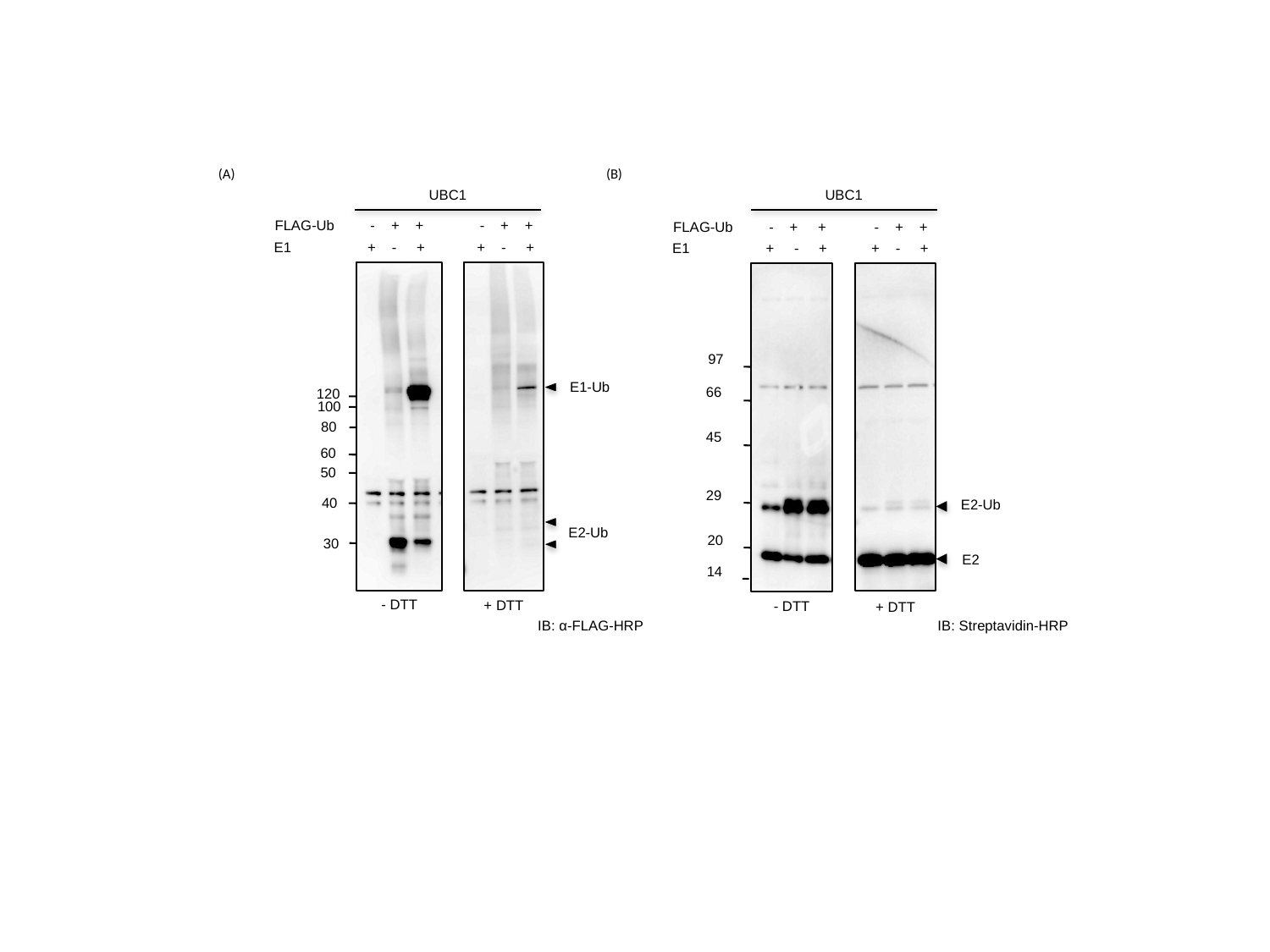

(A)
(B)
UBC1
UBC1
FLAG-Ub - + + - + +
FLAG-Ub - + + - + +
E1 + - + + - +
E1 + - + + - +
97
E1-Ub
66
120
100
80
45
60
50
29
E2-Ub
40
E2-Ub
20
30
E2
14
- DTT
+ DTT
- DTT
+ DTT
IB: α-FLAG-HRP
IB: Streptavidin-HRP

Supplement: Additional file 1: — Wheat germ-based thioester assay of UBC1. Wheat germ-based thioester assay of UBC1. Bio-UBC1 crude protein was analyzed as a representative E2 in the presence or absence of FLAG-Ub and/or E1. The reactions incubated for 2 h at 37 °C and treated with DTT or 8 M urea (-DTT) (a) Immunoblot analysis with anti-FLAG-HRP antibodies show the presence of DTT-sensitive Ub conjugation activity for UBC1 regardless the addition of E1 (as referred by the two bottom arrows). (b) Immunoblot analysis with streptavidin-HRP detected the bio-UBC1 (as referred by the lower arrow) and showed DTT-sensitive band shift equivalent to single Ub adduct (as referred by the top arrow). (PPTX 198 kb) [file 12870_2015_660_MOESM1_ESM.pptx]
