# Supplementary material for: Wheat germ-based protein libraries for the functional characterisation of the Arabidopsis E2 ubiquitin conjugating enzymes and the RING-type E3 ubiquitin ligase enzymes
Source: BMC Plant Biol. 2015 Nov 10;15:275. doi: 10.1186/s12870-015-0660-9 (PMC4641371; doi:10.1186/s12870-015-0660-9)
Supplement: Additional file 2: — Flow chart of the wheat germ-based procedure for the production of Arabidopsis RING protein library. The first step involves the high-throughput preparation of DNA templates for transcription using 2 step “split-primer” PCR, followed by in vitro transcription using phage-coded SP6 RNA polymerase, and finally translation using wheat germ cell-free system. All the steps were carried out in 96-well microtiter plates. (PPTX 55 kb) [file 12870_2015_660_MOESM2_ESM.pptx]

## Slide 1
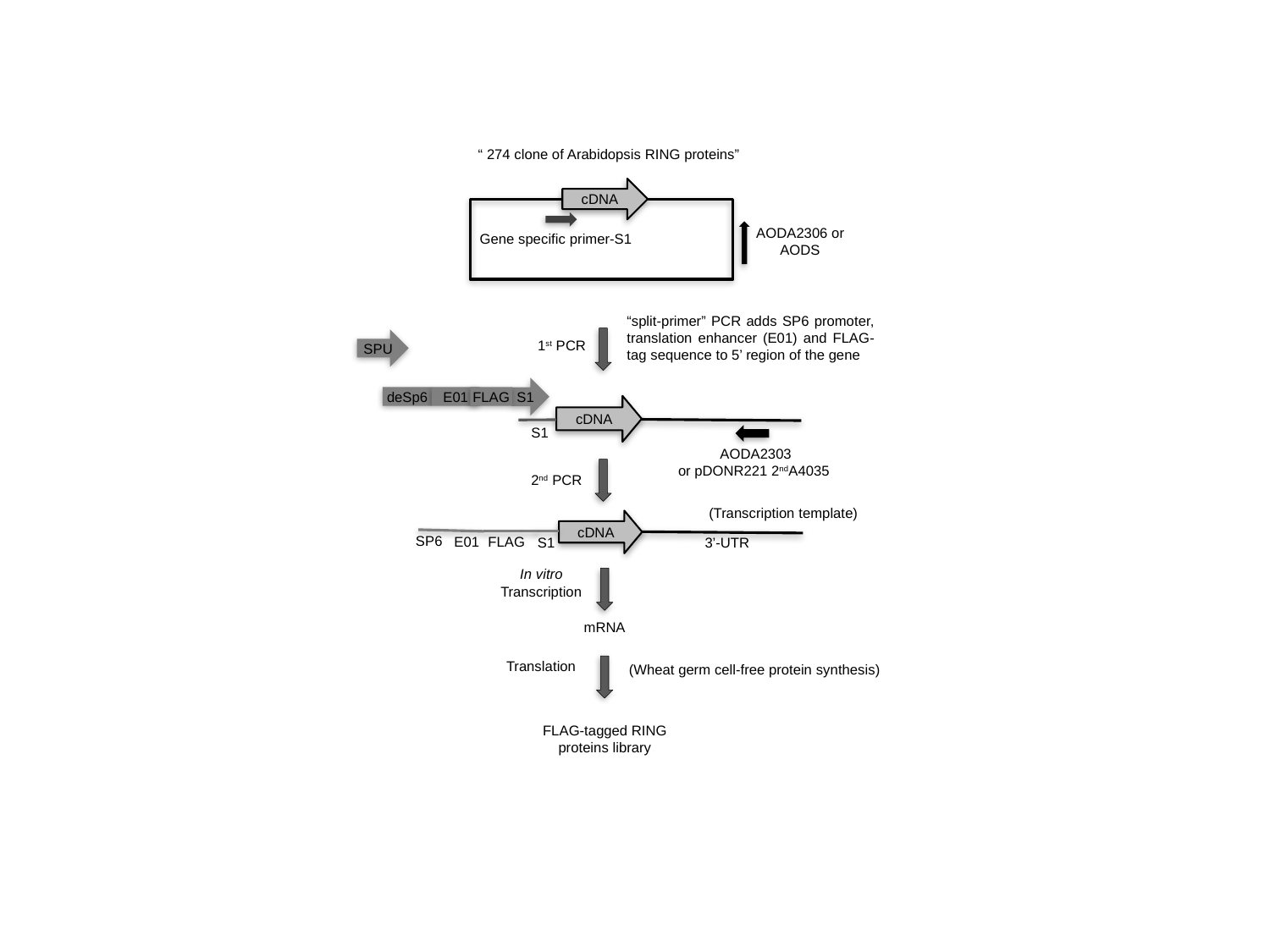

“ 274 clone of Arabidopsis RING proteins”
cDNA
AODA2306 or AODS
Gene specific primer-S1
“split-primer” PCR adds SP6 promoter, translation enhancer (E01) and FLAG-tag sequence to 5’ region of the gene
SPU
1st PCR
S1
deSp6
E01
FLAG
cDNA
S1
AODA2303
or pDONR221 2ndA4035
2nd PCR
(Transcription template)
cDNA
SP6
E01
FLAG
S1
3’-UTR
In vitro Transcription
mRNA
Translation
(Wheat germ cell-free protein synthesis)
FLAG-tagged RING proteins library
